# Advancements in Catalytic Depolymerization Technologies

**DOI:** 10.3390/polym17121614

**Published:** 2025-06-10

**Authors:** Goldie Oza, Fabrizio Olivito, Apurva Rohokale, Monica Nardi, Antonio Procopio, Wan Abd Al Qadr Imad Wan-Mohtar, Pravin Jagdale

**Affiliations:** 1 Centro de Investigation y Desarollo Tecnologico en Electroquimica Parque Tecnológico Querétaro, Queretaro CP 76703, Mexico; goza@cideteq.mx (G.O.); arohokale@cideteq.mx (A.R.); 2 Department of Environmental Engineering, University of Calabria, Via P. Bucci, 87036 Arcavacata di Rende, Italy; 3 Department of Health Sciences, University Magna Graecia of Catanzaro, Viale Europa—Campus Universitario S. Venuta—Loc. Germaneto, 88100 Catanzaro, Italy; monica.nardi@unicz.it (M.N.); procopio@unicz.it (A.P.); 4 Functional Omics and Bioprocess Development Laboratory, Institute of Biological Sciences, Faculty of Science, Universiti Malaya, Kuala Lumpur 50603, Malaysia; qadyr@um.edu.my; 5 Solar Research Institute (SRI), Universiti Teknologi MARA (UiTM), Shah Alam 40450, Malaysia; 6 Istituto Italiano di Tecnologia—IIT, Centre for Sustainable Future Technologies (CSFT), Via Livorno 60, 10144 Turin, Italy; pravin.jagdale@circular-carbon.com; 7 BHUMI—Bharat Harit Urja Managment and Innovations Pvt Ltd., Bansenstrasse, 21075 Hamburg, Germany

**Keywords:** circular economy, catalysis, sustainable chemistry, polymers

## Abstract

The increasing market demand and rising costs of raw materials have intensified interest in renewable and sustainable sources. As a result, the production of building-block chemicals from natural products or synthetic feedstocks has driven scientific research toward catalytic strategies for the depolymerization of these materials. Polymer chemistry offers significant opportunities for recycling, as polymer synthesis typically begins with monomeric units. Emerging non-destructive techniques now allow for the recovery of these original reagents. This review summarizes recent advances in catalytic methods for the depolymerization of polymers derived from both natural sources, such as cellulose and lignin, and synthetic sources, including conventional plastics. The review is structured in three main sections: catalytic depolymerization of cellulose, lignin, and plastics. Special emphasis is placed on recent studies that explore innovative methodologies. The raw materials obtained through these processes can be reintegrated into production cycles, contributing to the development of a fully circular economy.

## 1. Introduction

The overproduction of new materials has led to the exploitation of fossil resources at unprecedented levels, resulting in irreversible effects on ecosystems [1,2]. This depletion of building-block chemicals has contributed to rising market prices [3,4]. In recent decades, both academia and industry have increasingly invested in more sustainable resources and related methodologies to address these challenges [5,6]. Today, sustainability is recognized as a multidimensional concept encompassing not only economic aspects but also environmental considerations [7,8]. Polymers, both natural and synthetic, are among the most abundant materials on Earth [9,10]. In particular, biomass has garnered considerable attention over the past few decades, having been transformed from a natural resource or waste product into a valuable feedstock for the production of chemicals, materials, and energy [11,12,13]. Cellulose, a natural glucose polymer found in plants, has been extensively functionalized over the years to yield materials suitable for a wide range of applications [14,15,16]. Among the various research areas involving this polymer, its depolymerization remains one of the most extensively studied and compelling topics, continuing to attract substantial interest [17,18,19]. Depolymerization cleaves the polymer chain into glucose, the fundamental building block, triggering cascade reactions that generate what are now referred to as platform molecules [20,21,22]. These platform molecules have shown broad application potential and are emerging as viable and, in some cases, superior alternatives to their fossil-based counterparts [23,24,25]. Lignin, the other major component of biomass, was historically underutilized but is now recognized as a key resource with significant future potential [26,27,28]. This shift in perspective stems from its status as a largely untapped sustainable feedstock and its abundance as an industrial byproduct, particularly from the paper manufacturing sector [29,30,31]. Today, lignin is increasingly being explored as a renewable source of aromatic compounds, which are of great interest for the development of biofuels and value-added chemicals [32,33]. Although lignin is an aromatic polymer composed of cross-linked phenolic units derived from phenylpropane, with a structure that varies depending on plant type and other factors [34], it can also serve as a precursor to aliphatic molecules such as linear and cyclic alkanes, traditionally sourced exclusively from petroleum [35,36]. When harnessed sustainably, the biopolymers discussed thus far represent an inexhaustible resource with considerable untapped potential. Despite these advancements, fossil-derived raw materials remain widely used, largely due to economic considerations, including market demand and the well-established knowledge of the properties and applications of the resulting materials. Each year, over 380 million tons of polyethylene terephthalate (PET) are produced globally. PET, commonly found in everyday products, is primarily synthesized from terephthalic acid and ethylene glycol, both traditionally sourced from fossil feedstocks [37]. Other extensively used fossil-based plastics include polyvinyl chloride (PVC), polyethylene (PE), polystyrene (PS), polypropylene (PP), among others [38,39,40]. Contemporary research increasingly focuses on replacing fossil-derived chemicals with sustainable alternatives or developing new methods for their production [41,42]. In particular, chemical depolymerization using environmentally friendly catalysts and sustainable methodologies has emerged as a promising strategy to regenerate plastic monomers, enabling their reintegration into a circular economy, including applications such as food packaging [43,44,45].

This review is organized into three sections, each presenting recent innovations in catalytic depolymerization methods for cellulose, lignin, and widely used synthetic plastics. The selected studies illustrate approaches that integrate the principles of sustainable and green chemistry. Particular attention is given to the regenerated monomers, which not only enable the reproduction of original materials for reuse-based systems but also open avenues for alternative applications.

## 2. Catalytic Depolymerization of Cellulose

Cellulose is an insoluble polymer composed of D-glucose units linked by β-1,4-glycosidic bonds, forming a structure characterized by both crystalline and amorphous domains [46,47]. The extensive network of intra- and intermolecular hydrogen bonds gives rise to a highly cross-linked molecular architecture, which contributes to its pronounced insolubility in most solvents. This unique structural complexity also poses significant challenges for cellulose depolymerization. The sugars obtained from cellulose degradation serve as key precursors for the synthesis of various biofuels and value-added chemicals, including ethylene, ethanol, glycerol, lactic acid, glycol, and levulinic acid.

Several strategies have been developed to convert cellulose into glucose, among which enzymatic hydrolysis and catalytic hydrolysis, employing either liquid or solid acid catalysts, are the most extensively studied. Enzymatic hydrolysis is particularly attractive due to its mild reaction conditions and high selectivity, enabling the efficient release of fermentable sugars from diverse biomass sources. Notably, enzymatic approaches exhibit exceptional selectivity toward glucose. However, their broader industrial application is hindered by limitations such as high enzyme costs, challenges in catalyst recovery and recycling, and relatively low overall efficiency.

Mineral and organic acids, including H_2_SO_4_, HCl, acetic acid, and oxalic acid, have been widely employed as catalysts for the hydrolysis of cellulose [47,48]. However, the use of these acids presents several significant challenges: (i) difficulty in separating and recovering the acids from the sugar-rich hydrolysate, (ii) the generation of acidic wastewater requiring treatment, (iii) corrosion of processing equipment, and (iv) decreased sugar yields due to the degradation of monosaccharides into byproducts such as 5-hydroxymethylfurfural (HMF), furfural, and levulinic acid (LA) under high-temperature conditions [48,49].

To overcome these limitations, solid acid catalysts have emerged as promising alternatives for cellulose hydrolysis. In comparison to conventional liquid acids, solid acids facilitate easier separation, recovery, and reuse, thereby enhancing both the sustainability and overall efficiency of the process.

### 2.1. Solid Acid Catalyst Depolymerization of Cellulose

Solid acid catalysts play a pivotal role in the depolymerization of cellulose and the conversion of biomass into value-added chemicals and biofuels. Compared to homogeneous acids such as H_2_SO_4_ and HCl, solid acids are favored for their recyclability, lower corrosiveness, and enhanced selectivity in product formation. Representative examples include Amberlyst 15, 35, and 70, as well as Nafion and zeolite Y, which have demonstrated particular effectiveness in facilitating cellulose hydrolysis within ionic liquid media. Amberlyst 15 DRY, a sulfonated polystyrene resin, is especially efficient in hydrolyzing cellulose into glucose due to its strong Brønsted acidity and excellent reusability. Moreover, Amberlyst resins exhibit good stability in acidic anion-based ionic liquids, whereas they degrade rapidly in basic anion systems such as 1-butyl-3-methylimidazolium acetate.

Zeolites, such as HZSM-5, offer both Brønsted and Lewis acid sites, making them suitable for a range of reactions, including hydrolysis and isomerization. Similarly, metal oxides like sulfated zirconia are valued for their thermal stability and tunable acidity, making them well-suited for high-temperature depolymerization processes [50,51,52]. Ionic liquids, such as 1-allyl-3-methylimidazolium chloride (AMIMCl), are capable of dissolving cellulose at concentrations up to 25 wt% [53]. Their unique solvation properties make ionic liquids particularly advantageous for cellulose depolymerization applications.

**Scheme 1:**
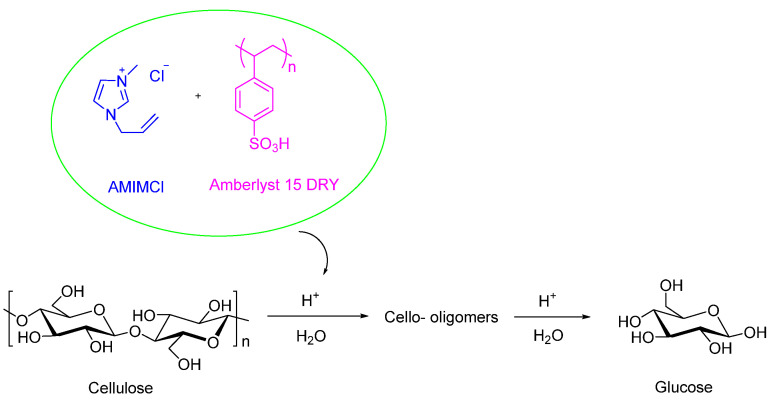
Mechanism of depolymerization of cellulose [49].

In a notable study, Pang et al. [49] investigated the combined use of ionic liquids and solid acid catalysts to process cellulose fibers derived from various agricultural byproducts, including sugarcane bagasse, sago pith, sawdust, and corn cobs. A 5 wt% cellulose solution was prepared by dissolving these fibers in 1-allyl-3-methylimidazolium chloride (AMIMCl). The addition of a small amount of Amberlyst catalyst initiated the depolymerization process via ion exchange with the ionic liquid, resulting in the in-situ generation of hydronium ions (H_3_O^+^). These protons played a critical role in cleaving the β-1,4-glycosidic bonds within the cellulose structure, leading to the formation of cello-oligomers along with reducing sugars and glucose as byproducts. This innovative approach offers a promising pathway for the efficient extraction of high-value components from lignocellulosic biomass (see Scheme 1).

The experiment demonstrated that the degree of polymerization by weight (DPw) of the resulting cello-oligomers was strongly dependent on the initial DPw of the cellulose fibers subjected to depolymerization. Specifically, cellulose fibers derived from corn cobs and sawdust, which had initial DPw values of approximately 351, yielded cello-oligomers with average DPw values of 88 and 72, respectively, after approximately 40 min of reaction time (Table 1) [49].

In contrast, cellulose fibers derived from sugarcane bagasse and sago pith, which exhibited higher initial average DPw values of 571 and 564, respectively, produced cello-oligomers with lower DPw values of 37 and 30 after extended reaction times of 60 and 100 min. Notably, the depolymerization of sago pith fibers proceeded at a faster rate than that of sugarcane bagasse. This accelerated depolymerization is likely due to a more uniform DPw distribution within the cellulose fibers of sago pith [49].

Although solid acid catalysts offer several advantages, their efficiency in cellulose hydrolysis is often limited by the high crystallinity of cellulose, which restricts catalyst access to the glycosidic bonds. This limited accessibility hampers effective glucose production, presenting a significant challenge for biorefineries reliant on sugar-based platforms. A promising strategy to overcome this barrier involves the pretreatment of cellulose using molten salt hydrates (MSHs) [54].

**Figure 1:**
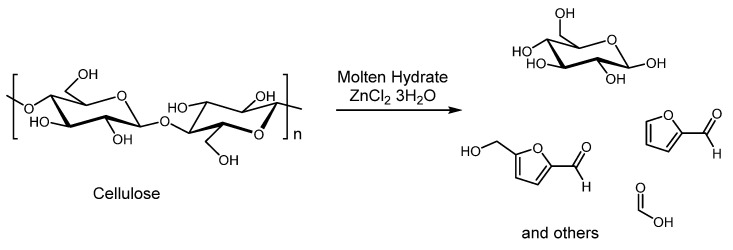
Methodology for screening cellulose catalytic conversions [55].

In a recent study, Paiva et al. [55] conducted cellulose conversion experiments using a Radleys Carousel Reactor. In a typical experimental setup (Figure 1), 0.05 g of cellulose was mixed with either 0.05 g or 0.025 g of catalyst in a solution containing 2.5 g of ZnCl_2_, maintaining a constant molar ratio of salt to water at 3.0.

The mixture was transferred to a reaction flask equipped with a magnetic stir bar and heated in the Radleys Carousel reactor at either 70 °C or 90 °C, with continuous stirring at 600 rpm for 2 to 5 h. Upon completion of the reaction, the flask was cooled in an ice bath. The catalysts were then separated by centrifugation, and 0.3 g of the resulting hydrolysate was diluted tenfold prior to product analysis [55].

Table 2 summarizes the conversion of cellulose using acidic molten salt hydrates (AMSHs) in the presence of various catalytic agents, highlighting the differences in product yields. Notably, compared to conventional inorganic acids such as H_2_SO_4_, lithium bromide (LiBr)-acidified MSH employed as a mesoporous solid hydrolysis catalyst at 85 °C yielded significantly higher amounts of glucose [56]. Furthermore, elevated temperatures in the presence of ZnCl_2_ facilitated substantial production of 5-hydroxymethylfurfural (HMF) [57]. When HZSM-5 (SiO_2_/Al_2_O_3_) was utilized at 90 °C for 2 h, the glucose yield was slightly lower than that obtained with the SO_4_^2−^/TiO_2_ catalyst [58].

Cellulose conversion using LiBr at elevated temperatures yielded variable glucose production depending on the catalyst and reaction conditions. Specifically, at 120 °C, Beta and ZSM-5 zeolites produced glucose yields of approximately 28%, whereas activated carbon at 110 °C achieved a significantly higher glucose yield of 80% [52,59]. At elevated temperatures (175 °C), catalysts such as NbOPO_4_, Nb_2_O_5_, NbOPO_4_/HZSM-5, and HZSM-5 facilitated the production of 85.1% levulinic acid (LA) alongside 5% 5-hydroxymethylfurfural (HMF) [59].

Romeo and co-workers [17] developed an innovative green process for converting microcrystalline cellulose into bio-oil and cellulose citrate via an open-air reaction catalyzed by molten citric acid. The molten carboxylic acid was able to penetrate the less accessible regions of the cellulose by disrupting the network of intra- and intermolecular bonds, thereby promoting simultaneous hydrolysis into bio-oil and esterification into cellulose citrate.

### 2.2. Mechanocatalytic Depolymerization of Cellulose

Mechanocatalytic depolymerization of cellulose integrates mechanical energy, such as ball milling, with catalytic activity to break down cellulose into smaller molecules. This approach disrupts the crystalline structure of cellulose, thereby enhancing its reactivity while reducing the overall energy input. Solid acid catalysts, including sulfonated carbon materials (e.g., CMK-3-SO_3_H) and perfluorinated ionomers (e.g., Aquivion), have been shown to significantly improve the efficiency of mechanocatalytic depolymerization. This method is environmentally friendly, minimizing the need for harsh chemicals and extreme reaction conditions. Key factors influencing the efficiency of this process include milling speed, catalyst characteristics, and moisture content [60,61].

**Figure 2:**
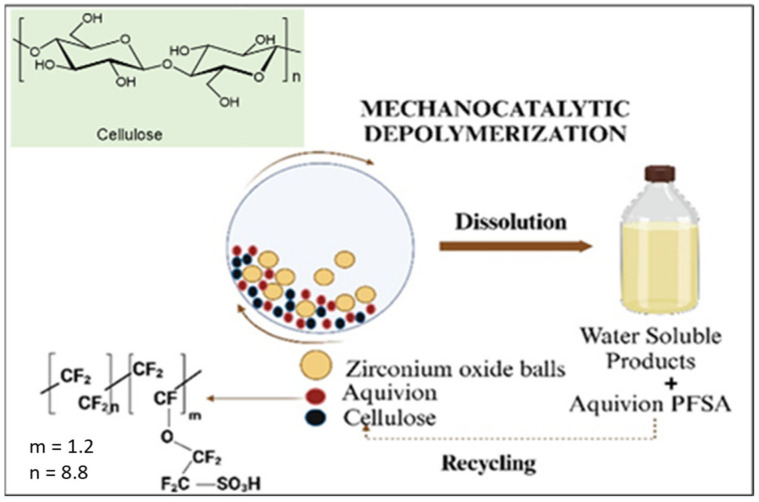
Mechanocatalytic depolymerization of cellulose results in the formation of water-soluble oligomers [62].

Zirconia-based catalysts, such as sulfated zirconia, demonstrate high acidity, excellent thermal stability, and resistance to deactivation, making them particularly effective in enhancing hydrolysis efficiency. These catalysts can be employed individually or in combination with other solid acids to improve cellulose breakdown. In the study by Karam et al., a planetary ball mill (Retsch PM 100) equipped with a zirconia grinding bowl was used [62]. Depending on the experimental conditions, zirconia, stainless steel, or tungsten carbide grinding balls with diameters of 2 mm, 3 mm, or 4 mm were employed. For effective grinding, a 125 mL zirconium oxide bowl was combined with twenty 10 mm zirconium oxide balls matching the bowl material (see Figure 2) [62].

Karam et al. subsequently removed the cellulose–catalyst milled mixture to assess the solubility of all milled samples. The solubility measurement consisted of three main steps: dispersion, filtration, and drying [62].

As reported in Table 3, the solubility data showed that microcrystalline cellulose (MCC) milled for 24 h under non-catalytic conditions exhibited less than 5% solubility [62]. In contrast, water solubility significantly increased when unmodified MCC subjected to planetary ball milling was directly treated with various solid acid catalysts. This enhancement suggests that the intrinsic acidic properties of the solid acid catalysts played a crucial role in breaking the β-1,4 glycosidic bonds in cellulose during the mechanocatalytic milling process [62].

According to Table 3, the high water solubility of microcrystalline cellulose (MCC) was achieved using the Aquivion PW98 catalyst, reaching approximately 90%, closely followed by the CMK-3-SO_3_H catalyst at around 87%. In contrast, the SBA-SO_3_H catalyst resulted in a lower solubility of approximately 60%, while kaolinite was the least effective, yielding only 50% water solubility [62].

Among the Aquivion catalysts tested, significant differences in performance were observed. Aquivion PW66 demonstrated the highest water solubility, approximately 99%, followed by PW98 at 90%, PW87 at 80%, and PW79 at 32%. Further studies explored the impact of ball milling duration on the catalytic efficiency of Aquivion PW66 and PW98, which were the most effective catalysts in the initial MCC depolymerization. The results indicated that extended milling times substantially improved water solubility, reaching 90% with Aquivion PW98 and an exceptional 99% with PW66 after 24 h [62].

Table 4 summarizes detailed information on the types and specifications of grinding balls employed in various applications as reported in the literature [61,63].

The first report in the scientific literature on the mechanocatalytic degradation of cellulose demonstrated that effective depolymerization occurs when the solid material is processed in a dry state. Using a high-energy shaker mill, the addition of kaolinite, a clay mineral, resulted in significant yields of water-soluble products, including glucose, fructose, and anhydroglucose (levo-glucose). This innovative approach highlights the potential of mechanochemical reactions for synthesizing valuable sugars from cellulose [63].

In their study, Hick et al. proposed that ball milling induces the delamination of layered compounds, thereby exposing surface acid sites. Although detailed product distributions of the resulting monosaccharides and oligomers were not provided, the authors suggested that the layered structure and acidity of the solid additive play crucial roles in the efficient mechanochemical degradation of cellulose [63].

Another promising strategy involves the use of layered HNbMoO_6_, which is characterized by a unique layered structure and high acidity, factors that enhance its catalytic efficiency in various applications [61]. Furusato et al. demonstrated that HNbMoO_6_ effectively catalyzes the mechanochemical depolymerization of cellulose due to its strong acidity, favorable layered structure, and excellent catalytic performance in acid-catalyzed reactions such as hydrolysis, esterification, and dehydration. Notably, HNbMoO_6_ produced water-soluble sugars at a rate three times higher than kaolinite, achieving a high yield of 72% after complete conversion within 24 h. Table 5 summarizes the mechanocatalytic depolymerization results reported by Furusato et al. [61]

All monometallic oxides tested, including NiO, SiO_2_, TiO_2_, and Nb_2_O_5_, exhibited no catalytic activity. Among the two-dimensional materials, acid-treated kaolinite produced 4% water-soluble sugars, while montmorillonite yielded 3%. Notably, the layered acid catalyst HNbMoO_6_ demonstrated superior performance, achieving a 14% yield of water-soluble sugars in solvent-free conditions, outperforming all other solid catalysts tested. In contrast, the Mg-Al hydrotalcite catalyst was ineffective in this reaction. The USY zeolite showed moderate catalytic activity with a sugar yield of 3%, comparable to that of montmorillonite [61].

The study also highlighted the significant impact of the number of zirconia grinding spheres on the total sugar yield. When only one or two zirconia spheres were employed, the total sugar yield remained below 1%, likely due to insufficient collision frequency as the spheres primarily moved along the vessel walls. Increasing the number of spheres to more than four enhanced the mechanical energy imparted through more frequent collisions, resulting in sugar yields between 12% and 14%. The highest water-soluble sugar yield of 72% was achieved after 24 h of grinding at 800 rpm and room temperature using layered HNbMoO_6_, corresponding to a 99% cellulose conversion based on weight loss. This yield was substantially higher than the 1% yield observed in the absence of any catalyst [61].

In a recent study, Yu et al. applied an acid-assisted mechanocatalytic depolymerization technique for the pretreatment of rice straw, which induced significant structural modifications in cellulose. Compared to untreated rice straw, the pretreated material exhibited markedly reduced particle sizes (from 279 to 11.8 µm), decreased cellulose crystallinity (from 43.05% to 22.71%), a 177% increase in surface area, and a 75% increase in surface oxygen relative to carbon content [64]. Enzymatic hydrolysis conducted over 12 h on the pretreated straw resulted in a total sugar yield exceeding 95%, significantly outperforming untreated straw (36.24%) as well as acid-impregnated (45.20%) and ball-milled straw (73.25%). These results underscore the effectiveness of acid-assisted mechanocatalytic depolymerization as a pretreatment method to enhance enzymatic hydrolysis and overall biomass conversion efficiency [64].

## 3. Catalytic Depolymerization of Lignin

**Figure 3:**
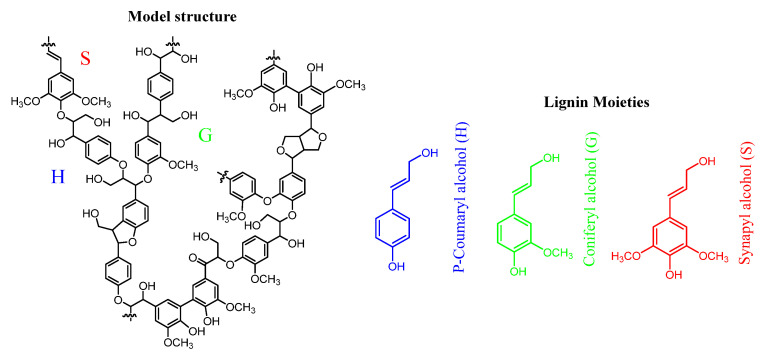
A segment of macromolecular lignin structure with its H, G, and S moieties. The figure referred from [68].

Lignocellulose is composed of various constituents, including lignin, hemicellulose, cellulose, proteins, and oils [65]. Lignin consists of phenylpropanoid units cross-linked through ether and carbon–carbon bonds (see Figure 3). Historically regarded as a low-value byproduct, lignin’s market value reached approximately USD 836.8 million in 2023 and is projected to grow at an annual rate of 6% between 2024 and 2032. Despite this, only a small fraction (1–2%) of lignin is currently valorized [66]. The complex, polyaromatic structure of lignin makes it a highly promising precursor for the synthesis of high-value chemicals and advanced materials [67].

Structurally, lignin adopts a helical configuration and is polymerized from three primary phenylpropane monomers: p-coumaryl alcohol (H), coniferyl alcohol (G), and sinapyl alcohol (S) (see Figure 3). These phenolic units are interconnected by a variety of ether and carbon–carbon linkages. The predominant linkage is the β-O-4 bond, which accounts for approximately 45% to 60% of the lignin structure. Other significant linkages include β-5 and 5-5, which together represent 5% to 25% of the polymer network. The selective cleavage of β-O-4 linkages is critical for efficient depolymerization of lignin into its constituent phenolic monomers [68].

Lignin valorization into value-added products has garnered significant interest in recent years. However, its complex and recalcitrant structure typically necessitates harsh depolymerization conditions, often involving high temperatures (~500 °C) and elevated pressures (~200 bar). In contrast, catalytic depolymerization presents a more promising and sustainable approach by enabling the selective cleavage of polymeric aromatic rings into smaller, valuable fractions under milder conditions [69,70].

### 3.1. Challenges and Opportunities

Lignin, a complex and recalcitrant biopolymer, is a major constituent of plant cell walls and a significant renewable source of aromatic compounds. Its heterogeneity and irregular structure, arising from the random polymerization of phenylpropanoid units, complicate selective bond cleavage [71]. The presence of various C–C and C–O linkages demand highly specific catalytic strategies, often resulting in energy-intensive and inefficient depolymerization processes [72]. Additionally, structural variations among lignin types—such as softwood, hardwood, and grass lignin—necessitate tailored depolymerization approaches, further complicating large-scale applications [73]. Despite these challenges, recent advances have opened promising avenues for efficient lignin depolymerization. Catalytic hydrogenolysis, oxidative depolymerization, and biocatalytic methods are among the most extensively explored techniques [71]. Metal-based catalysts containing Ru, Pd, or Ni have shown significant potential in selectively breaking down lignin into valuable monomers [74]. Moreover, engineered microbial pathways and enzyme-based strategies are gaining traction due to their ability to enable mild and sustainable lignin valorization [72]. Advances in solvent-based fractionation techniques, including deep eutectic solvents and ionic liquids, also offer promising routes to enhance lignin solubility and reactivity [75]. Integrating lignin depolymerization into existing biorefinery processes is critical for advancing a sustainable bioeconomy. Future research should prioritize catalyst optimization, scalable biotechnological development, and improved process economics to make lignin valorization commercially viable. Overcoming these challenges will position lignin as a valuable feedstock for bio-based chemicals, materials, and fuels, thereby supporting a circular bioeconomy [71,74].

Photocatalysis is one of the most promising sustainable strategies. Luo et al. [76] developed carbazolic porous organic frameworks (POFs) with tunable redox potentials to serve as efficient visible-light photocatalysts for the selective degradation of lignin β-O-4 model compounds. By fine-tuning the redox properties of these POFs, they achieved enhanced photocatalytic activity, leading to effective cleavage of C–O bonds under mild conditions. This approach underscores the potential of designing tailored photocatalysts for lignin depolymerization, contributing to sustainable biomass valorization strategies.

Nguyen et al. [77] present a catalytic, light-driven, redox-neutral method for the depolymerization of native lignin biomass at ambient temperature. The process involves proton-coupled electron-transfer (PCET) activation of alcohol O–H bonds to generate alkoxy radical intermediates, which promote β-scission of adjacent C–C bonds. This single-step depolymerization is driven solely by visible light, avoids stoichiometric reagents, and produces no stoichiometric waste. The method shows high efficiency and selectivity for cleaving the β-O-4 linkages in lignin, even amidst various PCET-active groups, and is also effective on β-1 linkages in model lignin dimers. These findings demonstrate the potential of visible-light photocatalysis for direct lignin biomass valorization into valuable aromatic feedstocks.

#### 3.1.1. Homogeneous Acid Catalysis

**Figure 4:**
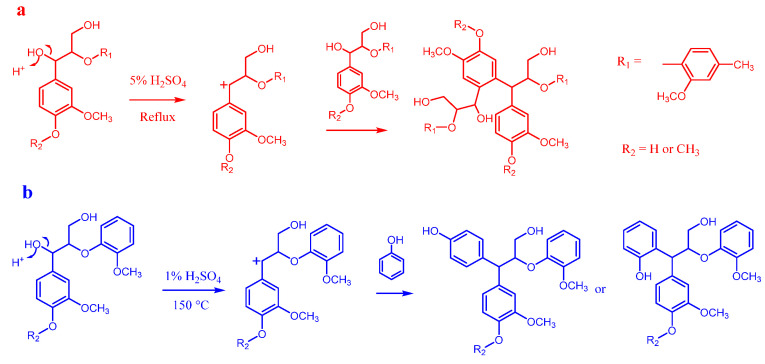
Typical reaction mechanism of lignin under acidic conditions. (**a**) Self-condensation between lignin molecules; (**b**) reaction between lignin and phenol [78].

Strong acids, such as hydrochloric acid, sulfuric acid, p-toluenesulfonic acid, and phosphoric acid, are commonly employed in homogeneous lignin depolymerization (see Figure 4) [78].

Strong acids such as hydrochloric acid, sulfuric acid, p-toluenesulfonic acid, and phosphoric acid are effective in lignin depolymerization due to their ability to attack ether bonds, particularly the prevalent β-O-4 and 4-O-5 linkages. This acid-catalyzed cleavage proceeds through protonation of the ether oxygen, forming reactive carbocations that undergo rearrangements and result in a complex mixture of phenolic compounds [79]. The efficiency of this process depends heavily on reaction parameters, including temperature, acid concentration, reaction time, and solvent choice, all of which influence product distribution and yield [70]. Increasing acid concentration accelerates protonation and nucleophilic attack rates, while elevated temperatures lower activation energy and promote faster lignin breakdown. However, high temperatures and prolonged reaction times also favor side reactions, producing undesirable byproducts. Thus, careful optimization is essential to maximize depolymerization efficiency and selectivity while minimizing secondary reactions [80].

Despite their high catalytic activity and relatively low cost, homogeneous acid catalysts face significant drawbacks. Their low selectivity leads to complex mixtures containing phenolic monomers, dimers, oligomers, and other degradation products. The harsh reaction conditions and corrosive nature of strong acids pose challenges in catalyst recovery, equipment durability, and product purification, complicating process sustainability and eco-friendliness [70,81]. Moreover, the corrosive environment necessitates specialized, corrosion-resistant equipment and stringent safety measures, increasing operational costs. These limitations highlight the need for improved catalytic systems or alternative approaches to develop more sustainable lignin valorization processes [70].

#### 3.1.2. Homogeneous Base Catalysis

Base-catalyzed depolymerization is a widely utilized approach for extracting phenolic monomers from lignin, employing bases such as calcium hydroxide (Ca(OH)_2_), sodium hydroxide (NaOH), potassium hydroxide (KOH), and organic bases like tetramethylammonium hydroxide [82,83]. In this mechanism, hydroxide ions (-OH^−^) initiate depolymerization by deprotonating the phenolic hydroxyl groups (-C_6_H_5_OH), forming reactive phenoxide ions (-C_6_H_5_O^−^). These phenoxide ions then attack the β-carbon of ether linkages within lignin, generating aromatic aldehydes and ketones through cleavage of the β-O-4 bonds [84]. This method is favored due to its relatively high catalytic efficiency at moderate temperatures and milder reaction conditions compared to acid-catalyzed methods.

However, solvent choice plays a crucial role in the success of base-catalyzed lignin depolymerization. For example, conducting reactions in aqueous media with low base concentrations often results in limited depolymerization and negligible product yields, indicating that water is generally unsuitable for this process [85,86].

**Scheme 2:**
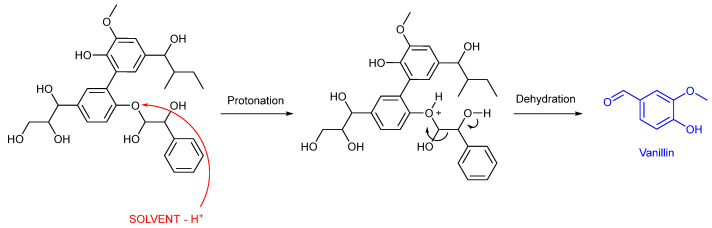
Reproduced from Biswas et al., “Effects of solid base catalysts on depolymerization of alkali lignin for the production of phenolic monomer compounds”, *Renewable Energy*, Vol. 175, pp. 270–280, © 2021, with permission from Elsevier [87].

A recent study by Bijoy Biswas and colleagues [87] demonstrated an effective depolymerization of alkali lignin using solid base catalysts, including CaO/CeO_2_, CaO/Al_2_O_3_, and CaO/ZrO_2_, at 180 °C. The researchers screened various solvents and found that ethanol and methanol facilitated bio-oil yields of approximately 50% when used with CaO/CeO_2_ and CaO/ZrO_2_ catalysts. Vanillin emerged as the predominant product of the catalytic liquefaction, attributed to the enhanced cleavage of β-O-4 linkages by the basic catalysts, which simultaneously increased bio-oil and vanillin yields (see Scheme 2). This approach underscores the potential of solid base catalysts in producing valuable phenolic compounds under relatively mild conditions.

##### Combined Acid-Base Catalysis

In recent years, combining acid and base catalysis has emerged as an effective strategy for lignin depolymerization. Xia Zhang and co-workers [88] developed an innovative procedure to depolymerize Kraft lignin into liquid fuels using a WO_3_-modified acid–base coupled hydrogenation catalyst. Efficient lignin conversion in such systems relies on the activation of both C–C and C–O bonds alongside the stabilization of reaction intermediates. In this study, the Lewis acid sites of WOₓ and TiO_2_, together with the basic sites of MgO, demonstrated synergistic effects by facilitating the adsorption, stabilization, polarization, and activation of lignin’s C–O and C–C bonds. Meanwhile, NiO sites played a crucial role in stabilizing intermediates through molecular hydrogen (H_2_) activation. Under selective hydrodeoxygenation conditions at 300 °C for 24 h, the process yielded 63.03% petroleum ether-soluble products and 21.56% monophenols. This approach offers promising perspectives for developing mild, sustainable, and efficient lignin conversion technologies.

#### 3.1.3. Homogeneous Metal Catalysis

The most commonly used catalysts in lignin depolymerization are metal catalysts due to their diversity, design flexibility, and outstanding catalytic efficiency. Various metals have been utilized, including transition metals, noble metals, and bimetallic catalysts, such as ruthenium (Ru), palladium (Pd), platinum (Pt), copper (Cu), nickel (Ni), and iron (Fe) [89,90,91]. Metal catalysts facilitate the hydrogenolysis reaction and activate hydrogen (H_2_), leading to the formation of aromatic monomers. They promote the cleavage of C–O and C–C bonds in lignin through multiple mechanisms, including hydrogenolysis, oxidation, and solvolysis:

- Hydrogenolysis involves the cleavage of bonds by the addition of hydrogen (H_2_), resulting in the formation of smaller molecules.
- Oxidation entails the removal of electrons from a molecule, leading to the formation of carbonyl groups (C=O) and other oxidized byproducts.
- Solvolysis involves the cleavage of bonds through a reaction with a solvent, producing smaller molecular fragments.

These mechanisms highlight the versatility and effectiveness of metal catalysts in lignin depolymerization [92]. Transition metal catalysts, often modified by ligands, can have their catalytic properties significantly tuned through electronic and steric effects [93]. The efficiency of these catalysts depends on multiple factors, including temperature, pressure, and solvent choice, all of which influence reaction rates and product selectivity [94]. Metal catalysts present several advantages, such as high catalytic activity and selectivity towards specific products. They are capable of operating under relatively mild conditions and can be supported on various substrates, enabling easier catalyst recovery and reuse. However, challenges remain, including the high cost of many metal catalysts and the need for elevated temperatures and pressures to achieve efficient depolymerization. Despite these limitations, their high efficiency and selectivity make metal catalysts promising candidates for lignin valorization [94].

**Scheme 3:**
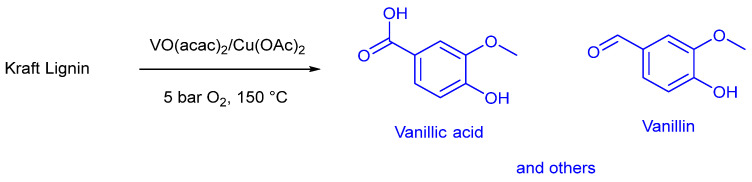
Oxidative lignin depolymerization into aromatic compounds [95].

In a recent study, Florian Walch and co-workers [95] demonstrated an effective oxidative depolymerization of Kraft lignin using a homogeneous vanadium–copper catalyst. Utilizing molecular oxygen as the oxidant, this system produced valuable aromatic monomers such as vanillin, vanillic acid, and acetovanillone, showcasing a promising route for selective lignin upgrading. The overall bio-oil yield reached 50% under optimized reaction conditions, with a yield in aromatic compounds of 27%. The representative scheme of the process is depicted in the following Scheme 3.

In a similar work, Omar Y. Abdelaziz and co-workers [96] developed an oxidative kraft lignin depolymerization using bimetallic V-Cu/ZrO_2_ catalysts. This zirconia-supported vanadium–copper catalyst proved to be efficient for the conversion of softwood LignoBoost Kraft lignin (LB), with a maximum monomer yield of 9% weight and a maximum selectivity in vanillin of 59%. Copper and vanadium were confirmed as promising homogeneous catalysts for this kind of application. We report a summary of homogeneous catalytic approaches for lignin depolymerization in the following Table 6.

#### 3.1.4. Heterogeneous Solid Acid Catalysis

Heterogeneous solid acid catalysts, including sulfonates, metal oxides, zeolites, heteropoly acids, and phosphates, are widely employed for lignin depolymerization due to their ability to promote cleavage of C–O bonds, leading to the formation of phenolic compounds [97,98,99]. These catalysts provide protons (H^+^) that initiate the reaction by protonating the ether oxygen in lignin (Lignin–O–R), thereby increasing the bond’s susceptibility to cleavage. This protonation facilitates C–O bond breakage, transferring the proton to oxygen and forming a hydroxyl group (-OH). The cleavage results in two primary products: a phenolic hydroxyl group (Lignin–OH) and a carbocation intermediate (R^+^). Depending on the nature of R, alkyl carbocations or protonated aromatic species form, which may subsequently undergo further transformations such as alcohol formation (R–OH) upon reaction with water or rearrangements [100,101].

A key advantage of solid acid catalysts lies in their regenerability; the proton is released after the reaction, enabling the catalyst to be reused for multiple cycles, enhancing both economic and environmental sustainability [102]. The β-O-4 ether linkage is particularly abundant in lignin, and its cleavage yields aromatic groups and highly reactive carbocation intermediates (R^+^ or CH_2_=Ar^+^), underscoring the effectiveness of solid acid catalysis in lignin depolymerization [103].

Selective depolymerization remains a major challenge, with catalysis playing a crucial role in improving selectivity. For example, Hongwei Ma et al. [104] reported the depolymerization of organosolv lignin catalyzed by non-noble nickel supported on zirconium phosphate (Ni/ZrP). Under conditions of 260 °C and 2 MPa H_2_ for 4 h using 15% Ni/ZrP-2.0, they achieved about 87% lignin conversion with less than 5% char formation. Phenolic monomer yields reached approximately 15%, with a notable amount of para-ethyl phenol produced, demonstrating the promising potential of this catalytic system for future lignin valorization [104].

The authors also evaluated the recyclability of the catalyst. The solid fraction obtained after hydrogenolysis was first calcined in air at 550 °C until a constant weight was reached. The resulting material was then reduced in a hydrogen atmosphere at 550 °C for 4 h. The regenerated catalyst was subsequently reused in a new reaction cycle to assess its performance upon recycling. The 15%Ni/ZrP-2.0 catalyst exhibits excellent reusability, maintaining a lignin conversion of 77.1% and a para-ethylphenol yield of 5.29 wt% after four consecutive runs. A slight weight loss of approximately 6 wt% was observed during each catalyst recovery cycle, likely due to the high viscosity of the solid residue composed of biochar and catalyst.

In another recent work, Zhuang Li and co-workers [105] developed a hydrogen-free strategy using solid acid catalysis to selective depolymerization of lignin into guaiacol. The authors hypothesized, supported by experimental evidence, the breaking of the C_ar_-C_α_ and C_β_-O bonds. In a 90% aqueous methanol solution, a guaiacol yield of 18.2% was demonstrated. The mechanism was hypothesized by combining experimental evidence with density functional theory. The authors highlighted how there is an interesting action of water, which promotes the decomposition of methanol to produce more reactive hydrogen species, thus favoring the C_ar_-C_α_ bond cleavage.

#### 3.1.5. Heterogeneous Metal-Supported Catalysis

Metal-supported catalysts consist of metal nanoparticles dispersed on various catalytic supports, enhancing catalyst dispersibility and modifying physicochemical properties to optimize catalytic performance. Their effectiveness arises from attributes such as high surface area, variable metal valencies, tunable acidity/basicity, structured porosity, well-defined topologies, and excellent thermal stability. Furthermore, these catalysts are cost-efficient and sustainable, making them ideal for lignin depolymerization applications. Common supports include silica (SiO_2_), activated carbon, alumina, and zeolites [106].

These catalysts exhibit high activity and stability, effectively facilitating the hydrogenolysis of lignin—a reaction that breaks down the complex polymer into smaller molecules, such as aromatic monomers. The general hydrogenolysis reaction can be summarized as

Lignin + H_2_ → Smaller molecules (e.g., aromatic monomers)

Mechanistically, both lignin and hydrogen molecules adsorb onto the catalyst surface, where the metal nanoparticles activate hydrogen molecules, increasing their reactivity. The activated hydrogen then cleaves the C–O and C–C bonds in lignin, producing smaller molecular fragments. These fragments subsequently desorb from the catalyst surface, regenerating the catalyst for further reaction cycles [102].

**Figure 5:**
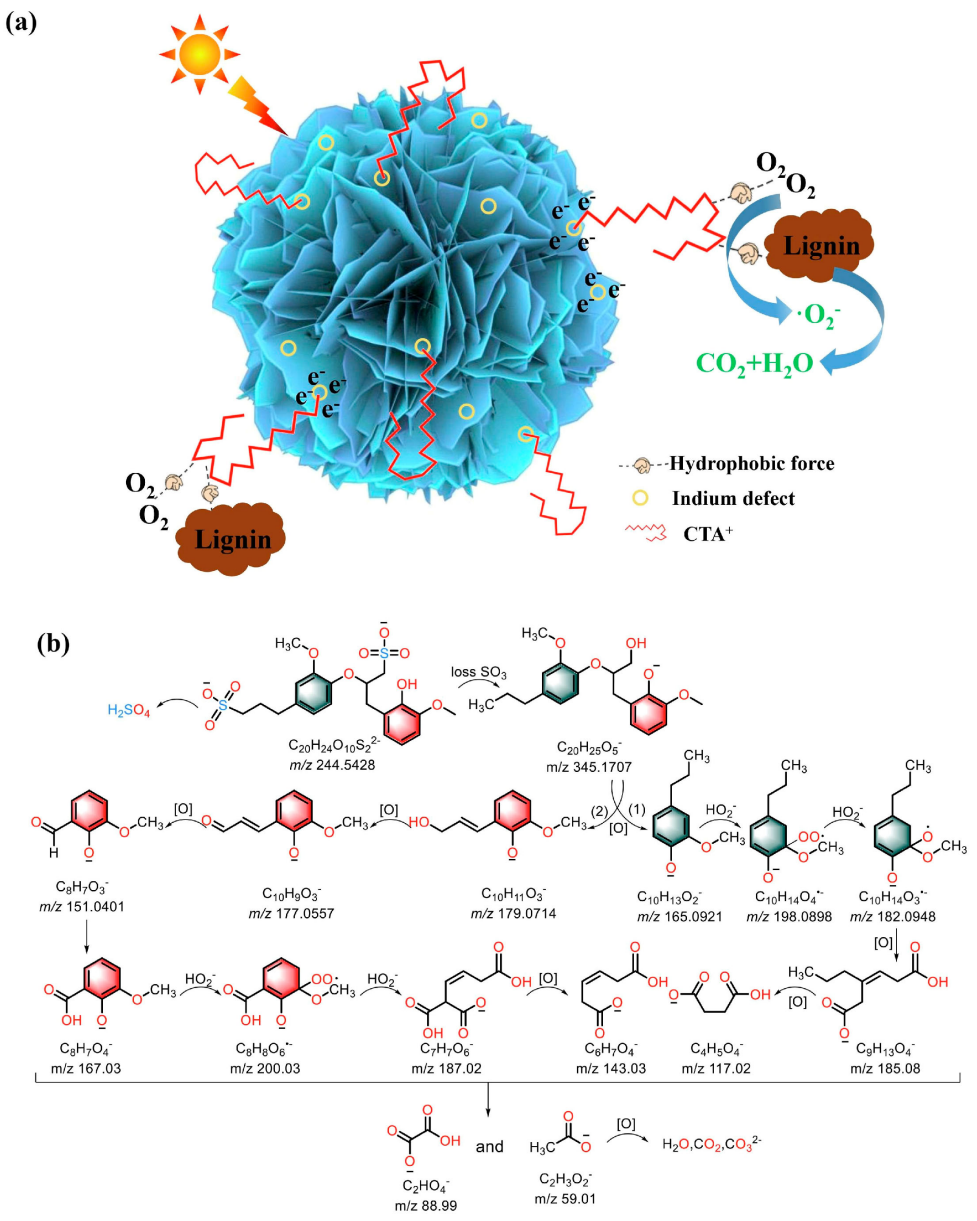
Schematic illustration of lignin degradation over the InₓS_3_-C catalyst (**a**) and the degradation pathway of lignosulfonate (**b**). Reproduced from Li et al., *Polymers*, 2024, 16(17), 2388. Licensed under CC BY 4.0. https://doi.org/10.3390/polym16172388 [108].

Chen et al. [107] developed a series of defective InₓS_3_-C catalysts by varying the ratio of indium chloride tetrahydrate to thioacetamide while incorporating hydrophobic CTA^+^ (cetyltrimethylammonium) ions at indium defect sites. The hydrophobicity of the In_x_S_3_-C materials could be finely tuned by adjusting both the defect concentration and the alkyl chain length of CTA^+^. The hydrophobic CTA^+^ facilitated the adsorption of both lignin sulfonate and oxygen, thereby improving mass transfer and promoting surface catalytic reactions. As a result, lignin was ultimately decomposed into low-molecular-weight compounds such as H_2_O and CO_2_. The underlying mechanism is illustrated in Figure 5.

The efficiency of lignin hydrogenolysis using metal-supported catalysts depends on several key factors. The type of metal plays a crucial role, as different metals exhibit distinct catalytic activities and selectivities toward specific bond cleavages in lignin. The loading and dispersion of nano-sized metal particles on the support directly influence the reaction rate and product selectivity, making the uniform decoration and distribution of metal on the support critical. The choice of support material also significantly affects metal nanoparticle dispersion and overall catalytic performance; inert supports are preferred to minimize unwanted side reactions. Furthermore, reaction parameters such as temperature, pressure, and reaction time substantially impact the efficiency and product distribution of lignin hydrogenolysis [109].

Overall, metal-supported catalysts provide a high surface area that promotes catalytic activity, while the support material stabilizes metal nanoparticles, preventing agglomeration and maintaining catalyst performance over multiple cycles. This combination of stability, recoverability, and reusability enhances the cost-effectiveness of metal-supported catalysts in lignin depolymerization processes [110,111].

Bijoy Biswas and colleagues [112] recently developed a notable method for the catalytic depolymerization of lignin into phenolic monomers using cobalt-supported calcium catalysts. Various reaction parameters, including reaction time, solvent type, temperature, and catalyst loading, were systematically evaluated. The optimal results were achieved using methanol as the solvent at 160 °C for 60 min, with a catalyst loading of 10 wt% Co/CaO. Under these conditions, the bio-oil yield reached 60.2% by weight. Notably, this catalytic system not only improved the overall bio-oil yield but also enhanced selectivity toward vanillin production, achieving a selectivity of 58.7%.

In a related study, Yanfang Zhu and co-workers [113] explored the effects of reaction conditions on lignin depolymerization catalyzed by metal-supported hydrotalcite catalysts. Pyrolysis without a catalyst at 500 °C for 60 min produced a bio-oil yield of 32.4% by weight. The introduction of a cobalt-supported hydrotalcite catalyst (Co/MgAl_2_O_4_) increased the bio-oil yield to 47.4% by weight. Additionally, the catalyst demonstrated selectivity toward G-type phenolic monomers, highlighting its effectiveness in cleaving lignin ether bonds (C–O–C).

The lignin-first approach is a well-established method for solubilizing lignin from lignocellulosic biomass by stabilizing reactive intermediates, which can be achieved through solvolysis, catalysis, or protecting group chemistry [114]. Shihao Su and co-workers [115] developed an innovative lignin-first depolymerization process using carbon nanotube-supported ruthenium catalysts to convert lignin into monophenols. Their study demonstrated that the efficiency of lignin hydrogenolysis into aromatic monomers strongly depends on the lignin source, with yields decreasing from hardwoods to softwoods, likely due to side condensation reactions. Additionally, catalyst recycling showed excellent performance retention, suggesting the practical viability of this catalytic system. This work opens promising avenues for the efficient conversion of lignin into valuable chemical compounds.

We report, in Table 7, a summary of heterogeneous catalysis for lignin depolymerization.

#### 3.1.6. Enzyme Catalysis

Enzyme catalysis plays a pivotal role in lignin depolymerization by offering a sustainable and environmentally friendly alternative to traditional chemical methods. Unlike conventional approaches, enzymatic depolymerization operates under mild conditions, ambient temperature, and neutral pH, using biocatalysts derived from renewable resources. Typically, this process utilizes oxygen or hydrogen peroxide as oxidants, often producing water as the only byproduct, thereby adhering to green chemistry principles. Among the enzymes employed, laccases and peroxidases are the most prominent due to their high specificity for cleaving particular bonds within the lignin structure. Laccases primarily target phenolic subunits and ether linkages, while peroxidases extend their activity to non-phenolic linkages, broadening the scope of lignin structures that can be effectively depolymerized [116].

Due to lignin’s heterogeneous and complex structure, defining precise chemical equations for its enzymatic depolymerization remains challenging. The reaction pathways depend heavily on the specific enzyme used, such as laccase or peroxidase, and the presence of mediators that can enhance the reaction. Enzymes often act synergistically, combining biological and chemical mechanisms to convert lignin into valuable products [117].

**Scheme 4:**
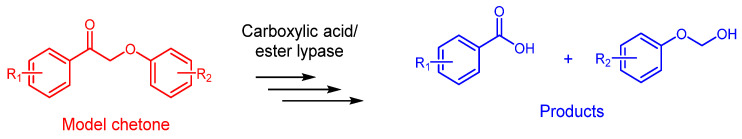
Depolymerization of lignin model compounds depolymerized by mediator–enzyme system [118].

In a recent study, Huan Zhang and co-workers [118] developed a methodology for lignin depolymerization utilizing mediator–enzyme catalysis. They employed various ketones as lignin model compounds to simulate C–C bond oxidation analogous to the β-O-4 linkages in lignin. The enzyme Novozym 435 was used as the catalyst for C–C oxidation, with hydrogen peroxide serving as the oxidant in an ethyl acetate solvent. This approach yielded excellent results across different model compounds, and the reaction pathway is illustrated in Scheme 4.

In another study, Justine Dillies and colleagues [119] reported an industrially relevant strategy for lignin depolymerization using laccase. They worked with different lignin types, organosolv lignin, Kraft lignin, and sodium lignosulfonate, in a solvent mixture of 1,4-dioxane and water. Laccase from Trametes versicolor was employed, with 25% 1,4-dioxane added to enhance lignin solubility in the reaction medium. The process showed excellent performance at 50 °C, with enzymatic activity maintained even after several days. Future research will focus on detailed product characterization to better understand the outcomes of this depolymerization technique.

**Advantages of Enzyme Catalysis**

- Mild Reaction Conditions: Enzymatic depolymerization occurs at ambient temperature and neutral pH, reducing energy requirements.
- Specificity and Selectivity: High specificity minimizes unwanted side reactions, yielding a more controlled product distribution compared to chemical methods.
- Environmental Friendliness: It avoids the use of harsh chemicals and produces minimal waste, supporting sustainable practices [83].

**Challenges and Limitations**
Despite its advantages, enzyme catalysis faces several challenges:

- Sensitivity to Reaction Conditions: Enzymes are sensitive to temperature, pH, and the presence of inhibitors, necessitating careful control of reaction conditions.
- Limited Lignin Accessibility: The complex structure of lignin can hinder enzyme accessibility, reducing depolymerization efficiency.
- Cost and Production Efficiency: The high cost of enzyme production impacts the economic viability of the process [120].

**Strategies for Improvement**
To overcome these challenges, research is focused on the following:

- Engineering more efficient enzymes with enhanced stability and activity.
- Developing cost-effective production methods.
- Implementing pretreatment strategies to improve lignin accessibility [120].

By addressing these limitations, enzyme catalysis holds significant potential for sustainable lignin valorization, paving the way for bio-based chemicals and materials within a circular bioeconomy.

## 4. Catalytic Depolymerization of Plastics

### 4.1. Depolymerization of Polyester Plastics

**Figure 6:**
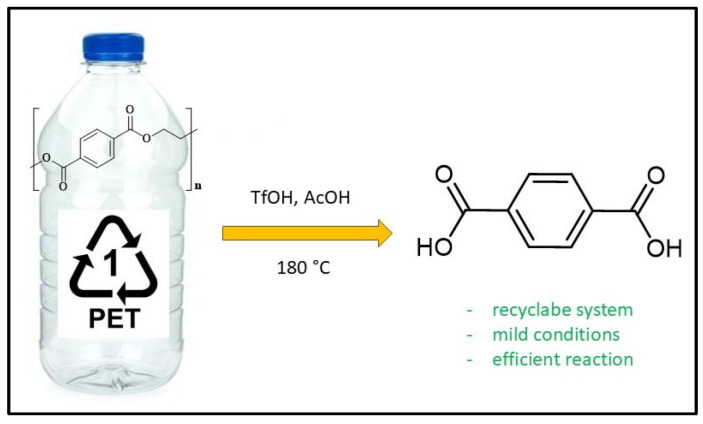
A schematic representation of the reaction process starting from PET [124].

Polyester plastics are among the most widely used due to their ester bonds (-C=O-OR), which confer advantageous properties such as processability, versatility, ease of production, chemical and bacterial resistance, and a high recyclability rate. Current research is focused on developing innovative strategies to recover starting monomers suitable for producing new plastics that maintain their original structural integrity [121,122,123]. In a recent study, Yu-Ji Luo et al. [124] proposed a closed-loop depolymerization process for polyesters that yields reusable monomers. This approach employs trifluoromethanesulfonic acid or metal triflates as recyclable catalysts combined with carboxylic acid and water (see Figure 6).

Before optimizing the reaction, the homogeneous acidolysis of 4-chlorobenzoate was studied using various Lewis and Brønsted acid catalysts in acetic acid. Water was found to play a crucial role in enhancing the reaction efficiency. Specifically, an initial yield of 49% was achieved within 5 min using 15 mol/mol trifluoromethanesulfonic acid (TfOH) at 180 °C, which increased to 78% upon adding 0.9 wt% water under the same conditions. The effectiveness of Lewis acids was also demonstrated; for example, the use of 15 mol% hafnium trifluoromethanesulfonate (Hf(OTf)_4_) resulted in a 90% reaction yield. These optimized conditions were then applied to the acidolysis depolymerization of PET as a model substrate, producing recycled terephthalic acid (rTPA) and glycol diacetate (EGDA) as the main products. Key results are summarized in Table 8.

**Figure 7:**
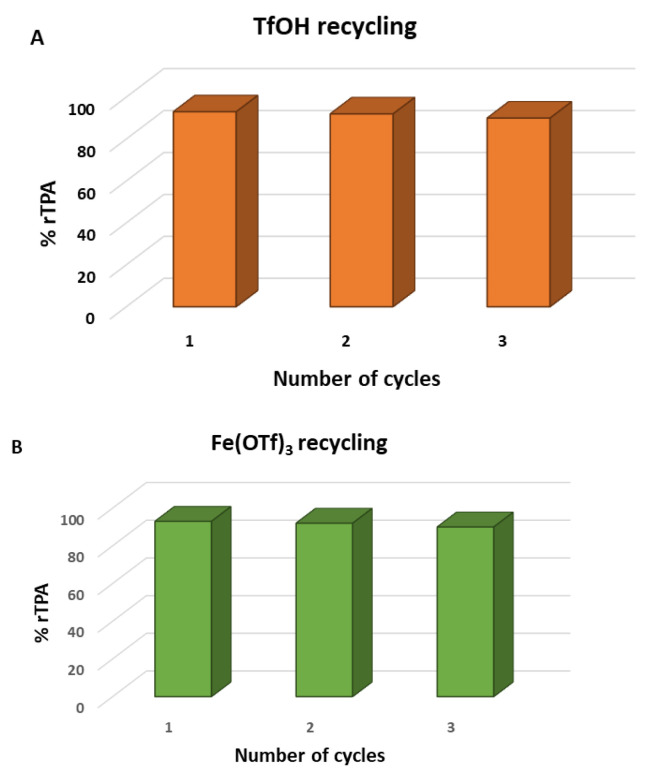
Recycling efficiency of (**B**) the Lewis acid catalyst [Fe(OTf)_3_] and (**A**) the Brønsted acid catalyst (TfOH) [124].

The reaction using Hf(OTf)_4_ without added water achieved a satisfactory yield of 60%, which increased to 72% upon the addition of 0.5 M water. In contrast, when other Lewis acidic transition metals, such as iron and aluminum triflates, were employed under the same conditions, the yields dropped to 64% and 60%, respectively. Excellent results were also observed with Brønsted acids; specifically, trifluoromethanesulfonic acid (TfOH) yielded 98% under identical conditions, whereas trifluoromethanesulfonimide resulted in a lower yield of 58%. Figure 7 presents the recycling efficiency of the Lewis acid catalyst [Fe(OTf)_3_] and the Brønsted acid catalyst (TfOH), expressed as the percentage yield of recycled terephthalic acid (rTPA) over multiple cycles.

**Scheme 5:**
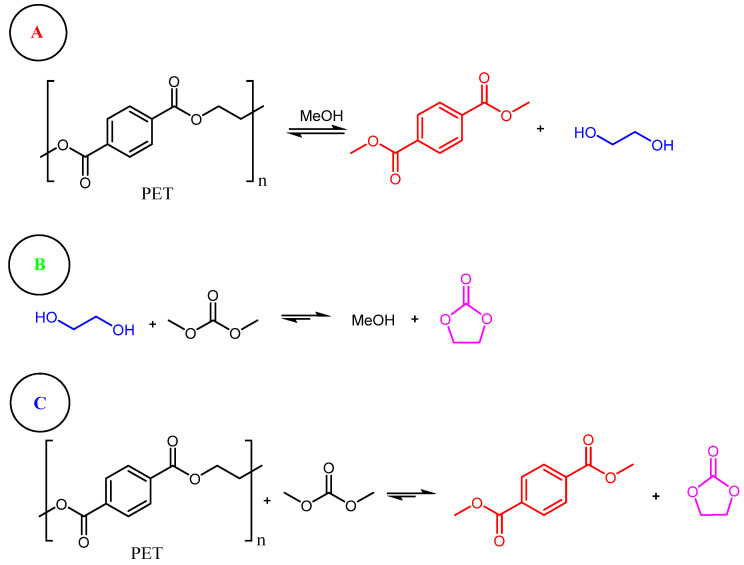
Adapted from Tanaka et al., *ACS Materials Au*, 2024, 4 (3), 335–345. © 2024 The Authors. Published by American Chemical Society under CC BY 4.0. Mechanism relative to the overall process divided into 3 reactions (**A**–**C**) [128].

As shown in Figure 7, the recycling of the catalytic system achieved recycled terephthalic acid yields of 90% after three cycles. To evaluate the recyclability of the catalytic system, both the acid catalyst (e.g., trifluoromethanesulfonic acid, TfOH) and metal triflate catalysts were recovered after each depolymerization cycle. The regeneration process involved the separation of the catalysts from the reaction mixture, followed by reuse without further purification. Notably, the hydrogenolysis step not only processed the depolymerized intermediates but also enabled the regeneration of the acid catalyst consumed during the acidolysis stage. The regenerated catalysts demonstrated excellent stability and activity over multiple cycles. Experimental results confirmed that both the catalytic performance and product yields remained nearly unchanged over repeated use, highlighting the robustness and sustainability of the closed-loop depolymerization system. These promising results pave the way for future studies in this field. Global fabric production is estimated at approximately 60 million tons per year, with polyethylene terephthalate being the predominant material used across various industries, including clothing and home furnishings [125,126,127]. In a study by Shinji Tanaka and co-workers [128], an innovative strategy for the depolymerization of polyester fibers was developed using dimethyl carbonate-aided methanolysis. This study focused on recycling polyester fibers primarily composed of polyethylene terephthalate. By employing methanolysis with dimethyl carbonate as a trapping reagent for ethylene glycol, the researchers achieved a low-temperature, efficient, and rapid depolymerization process. This method produced dimethyl terephthalate (DMT) with yields exceeding 90%. DMT, the diester form of terephthalic acid, is widely used in the production of recycled polyethylene terephthalate [128]. Conventionally, the methanolysis reaction is an equilibrium process that is challenging to perform under mild conditions. Scheme 5 illustrates the mechanistic pathways of the dimethyl carbonate-aided methanolysis process.

Methanolysis is a substitution reaction in which the ester’s alcoholic moiety is replaced by methanol, as illustrated in Scheme 5A for the depolymerization of PET. This reaction is a reversible equilibrium because the released ethylene glycol can react with the newly formed methyl ester, potentially reversing the process. To drive the reaction forward, the addition of dimethyl carbonate traps the ethylene glycol by converting it into ethyl carbonate, shifting the equilibrium towards the formation of depolymerization products, as shown in Scheme 5B. This approach effectively promotes PET depolymerization, as further depicted in Scheme 5C. Table 9 summarizes the key findings from the reaction condition optimization, highlighting the most influential parameters for the development of this method.

As shown in Table 9, NaOMe and KOMe exhibited higher catalytic activity compared to LiOMe, achieving yields of 95% versus 83%. Notably, NaOMe provided satisfactory yields even with a shorter reaction time of 1 h. Excellent results were also obtained using lower amounts of DMC and MeOH with a reaction time of 2 h.

**Figure 8:**
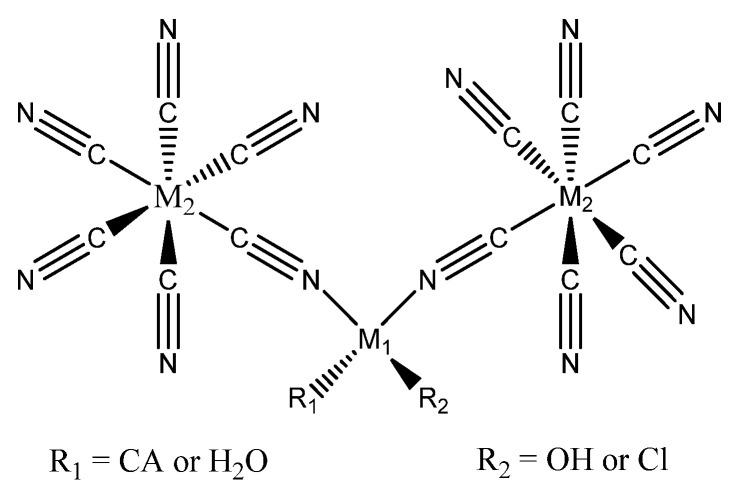
A schematic representation of the double metal cyanide (DMC) complex catalyst [131].

In a recent study, Xiaoshen Bai et al. [129] reported a solvent-free depolymerization of PET into dimethyl terephthalate (DMT) and ethylene glycol (EG) using heterogeneous catalysis, specifically through plastic–catalyst interfacial engineering. During reaction optimization, various metal acetates were screened for the depolymerization of PET microparticles sourced from plastic bottles. The experiments were conducted in closed vials with methanol at 100 °C for two hours. After testing several metal acetates, including Mn(OAc)_2_, Co(OAc)_2_, Ni(OAc)_2_, Cu(OAc)_2_, Zn(OAc)_2_, and Pb(OAc)_2_, a volcano-type activity plot was obtained [130]. Zinc acetate showed the most promising results after lead acetate, which was excluded due to toxicity concerns. SEM and TEM morphological studies suggested that the ZnAc_2_/MeOH interaction induces methanolysis of zinc acetate. This hypothesis was supported by the observation of zinc oxide nanocrystals on the plastic surface, likely acting as catalysts in the PET methanolysis process. The authors proposed that the significant catalytic activity at 140–200 °C is mainly due to ZnO_2_, while below 140 °C, the catalytic effect is attributed to ZnAc_2_. Notably, experiments using methanol vapor instead of liquid methanol demonstrated a solid–solid interaction between PET and zinc oxide at the interface, persisting until complete PET degradation due to the absence of interfering liquid solvent molecules. At 160 °C and after two hours, PET conversion was nearly quantitative in both ZnOAc with liquid methanol and in ethanol with ZnO vapors. This study presents new prospects for solvent-free depolymerization techniques for plastics. In another work, Tong Chang and co-workers [131] reported the interesting catalytic activity of the double metal cyanide complex (DMC) as a heterogeneous catalyst for polyester depolymerization into monomers and chemicals. Poly(ethylene terephthalate) (PET) was used as the reference plastic, and the most important reaction parameters, including the amount of ethylene glycol and the catalyst loading, were investigated. Figure 8 reports a schematic representation of the double metal cyanide (DMC) complex catalyst used in this study.

### 4.2. Depolymerization of Polyamides

Polyamides, particularly Nylon 6,6 and Nylon 6, are extensively utilized across industries such as textiles, automotive, construction, and medical sectors, reflecting significant global market demand [132]. These polymers are primarily petroleum-derived and are known for their high resistance to biodegradation and general decomposition, posing challenges to waste management and environmental sustainability [133].

**Scheme 6:**
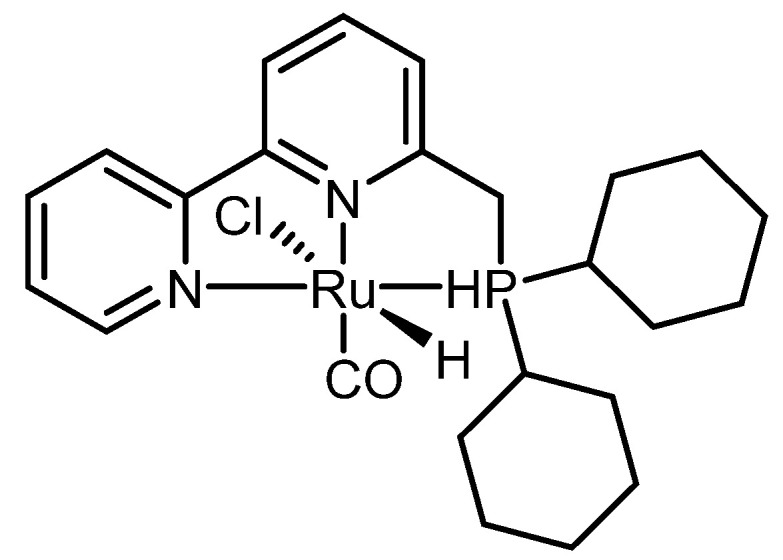
Ru pincer complex used to depolymerize low molecular weight polyamide [135].

Common disposal methods for polyamides include incineration and mechanical recycling; however, mechanical recycling often leads to the degradation of the material’s original properties [134]. In contrast, catalytic chemical depolymerization has emerged as a promising approach to recover the original monomers. For instance, Wei Zhou and co-workers [135] recently developed a catalytic hydrogenative depolymerization method for Nylon 6,6 using a ruthenium pincer complex, as illustrated in Scheme 6.

The authors began optimizing the catalytic system using low molecular weight polyamide 66 (Mw = 8240 g mol^−1^; amine end groups = 1748 mmol kg^−1^). The results are summarized in Table 10.

As shown in Table 10, high temperature plays a critical role in catalyst activation, with the highest monomer yields achieved at 200 °C. Hydrogen pressure is also a crucial parameter; optimal results were obtained at 100 bar, while lower pressures led to decreased yields of diamine and diol. After optimizing these reaction conditions, the method was successfully applied to Ultramid^®^ A 27, a commercial high molecular weight polyamide, resulting in diamine and diol yields of 19% and 18%, respectively.

**Figure 9:**
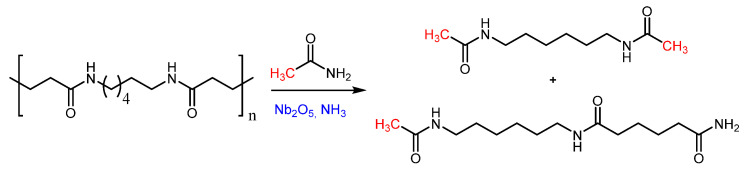
Adapted from R. Coeck and D. E. De Vos, *Chem. Commun.*, 2024, 60, 1444, under the terms of the Creative Commons Attribution-NonCommercial (CC BY-NC) licence (https://creativecommons.org/licenses/by-nc/4.0/, accessed on 6 June 2025). © The Royal Society of Chemistry [136].

In a complementary study, Robin Coeck and colleagues [136] developed a green and sustainable depolymerization approach for Nylon 66 via transamidation with a short primary amide catalyzed by niobium pentoxide (Nb_2_O_5_) and assisted by ammonia. Acetamide was selected as the model amide due to its bio-derived origin and biodegradability [137]. In this process, ammonia facilitates polymer cleavage through ammonolysis, as depicted in the reaction scheme in Figure 9.

**Scheme 7:**
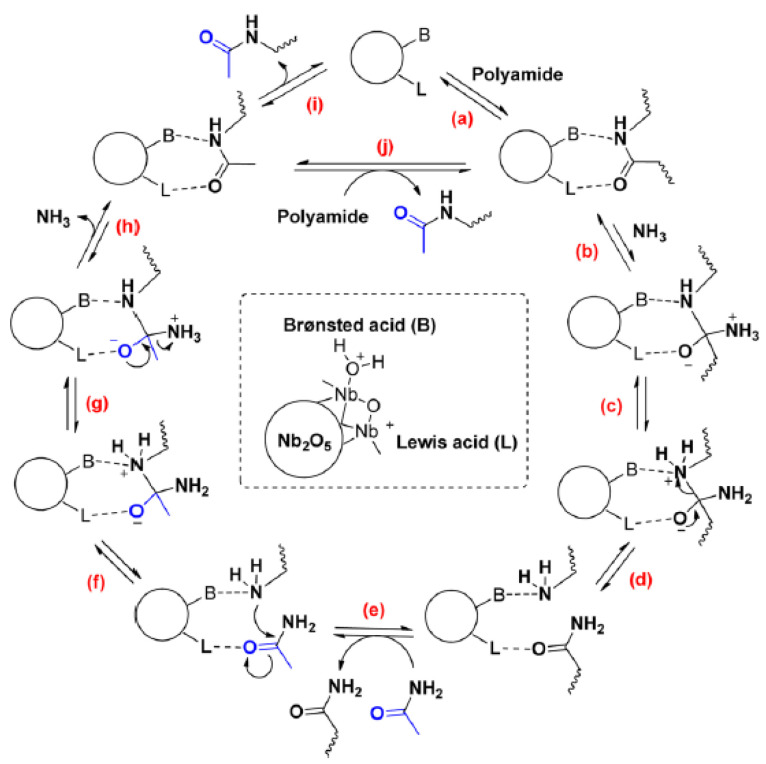
Reproduced from R. Coeck and D. E. De Vos, *Chem. Commun*., 2024, 60, 1444, under the terms of the Creative Commons Attribution-NonCommercial (CC BY-NC) licence (https://creativecommons.org/licenses/by-nc/4.0/). © The Royal Society of Chemistry [136].

For a better understanding, we report the scheme of the mechanism hypothesized by the authors in Scheme 7 [136].

As reported in Scheme 7, the carbonyl group of the secondary amide coordinates to the Lewis acid (L), while the nitrogen coordinates to the Bronsted site (B). The role of ammonia is fundamental because it carries out a nucleophilic attack on the amide adsorbed on the catalyst surface to form a primary amide and a free amino group [136].

At 225 °C and a reaction time of 16 h, Nylon 66 was fully depolymerized into monomers and dimers with yields of 94% and 5%, respectively. Using a maximum Nylon 66 concentration of 12.5 wt% in acetamide, valuable monomers such as N,N′-hexamethylene bis(acetamide), and adipamide were obtained, which have potential for various industrial applications.

**Scheme 8:**
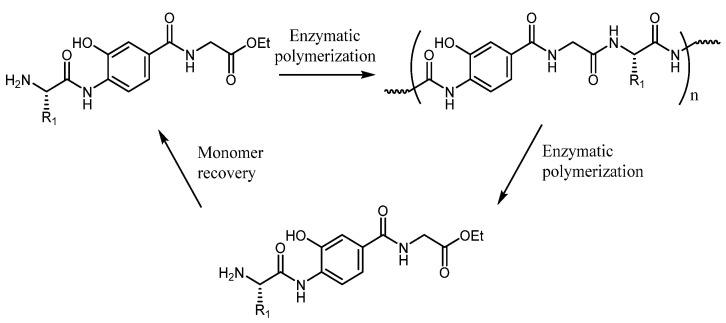
Chemoenzymatic polymerization/depolymerization of semiaromatic polyamides [138].

In another study, Kousuke Tsuchiya and co-workers [138] developed a novel method addressing both the polymerization and subsequent depolymerization of semiaromatic polyamides via enzymatic catalysis. Growing awareness of plastic waste issues has spurred research not only on creating new high-performance materials but also on their efficient disposal. Polyamides composed of 4-amino-3-hydroxybenzoic acid and peptide moieties were enzymatically synthesized through papain-catalyzed polymerization in aqueous media. These polymers were then selectively degraded back into their corresponding monomers upon treatment with proteinase K, as illustrated in Scheme 8.

Another innovative application of solid acid catalysis for polyamide depolymerization was reported by Yang Liu and colleagues [139]. They introduced the first sulfonated Fe-MOF catalyst specifically designed for depolymerizing polyamide 6 into water-soluble products. Remarkably, a complete conversion (100%) was achieved at 250 °C within just 1 h. Subsequent characterization using LC-MS confirmed that caprolactam and its oligomers were the primary products. Furthermore, a high selectivity of 90% towards caprolactam was attained after 5 h at the same temperature. These results highlight the potential for converting widely used polyamide materials into valuable, reusable monomers through sustainable catalytic processes.

### 4.3. Depolymerization of Polyurethanes

Polyurethanes are a versatile class of polymers distinguished by their unique combination of mechanical strength, flexibility, and chemical resistance [140]. They are typically synthesized through the catalytic reaction between diisocyanates (or polyisocyanates) and diols (or polyols), which forms urethane linkages (R–O–C=O–NH–R) [141]. In recent years, there has been growing interest in replacing traditional fossil-based reagents with sustainable, bio-based alternatives to produce eco-friendly polyurethanes [142]. These bio-based materials maintain high resistance and stability against environmental and biological degradation [143]. Aromatic polyurethanes remain among the most commonly used variants due to their exceptional durability and stability, making them suitable for diverse industrial applications [144]. However, their low environmental compatibility continues to present significant challenges, particularly in terms of sustainability and lifecycle impact during both production and use [145].

**Scheme 9:**
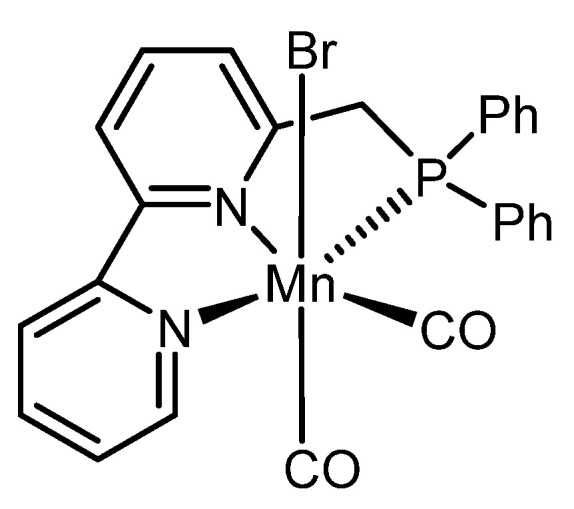
Mn pincer complex used to depolymerize polyurethanes [146].

Viktoriia Zubar and co-workers [146] discovered an innovative hydrogenative depolymerization method for aromatic polyurethanes using a manganese pincer complex (shown in Scheme 9) combined with high temperatures and suitable solvents. This approach enabled the efficient recovery of aromatic diamines and corresponding polyols in excellent yields.

**Figure 10:**
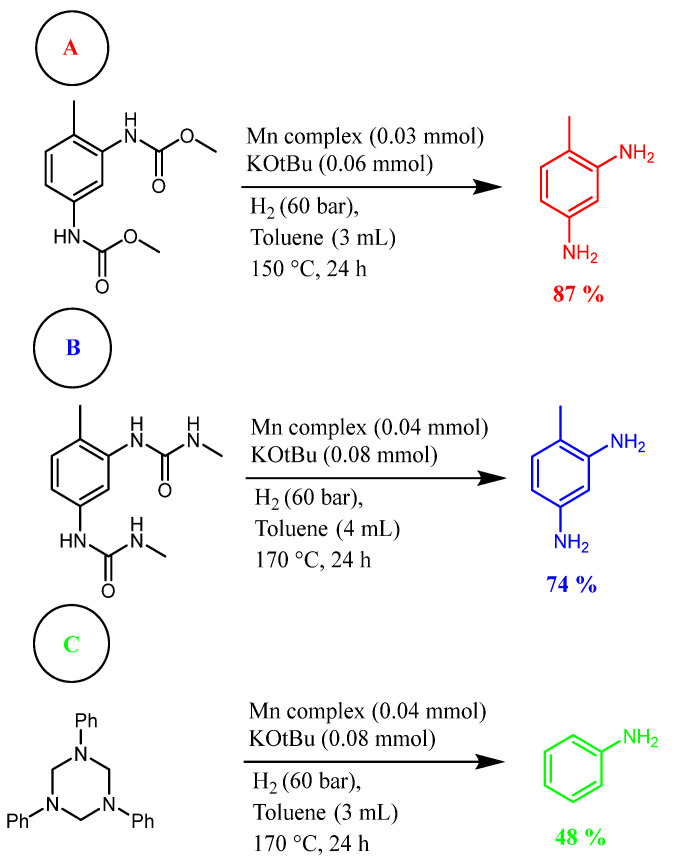
Catalytic depolymerization of three different polyurethanes (**A**–**C**) with the relative optimized reaction conditions and isolated yield.

Figure 10 shows three examples of different polyurethanes along with the corresponding optimized reaction conditions.

In the same study, the authors applied the optimized reaction conditions to commercial polyurethanes in addition to the self-made samples shown in Figure 10A–C. This was performed to assess whether additives like colorants and thickeners would affect the process yield. Surprisingly, depolymerization was achieved with conversion rates ranging from 66% to 100%, demonstrating the robustness of the method.

### 4.4. Depolymerization of Polyethers

**Scheme 10:**
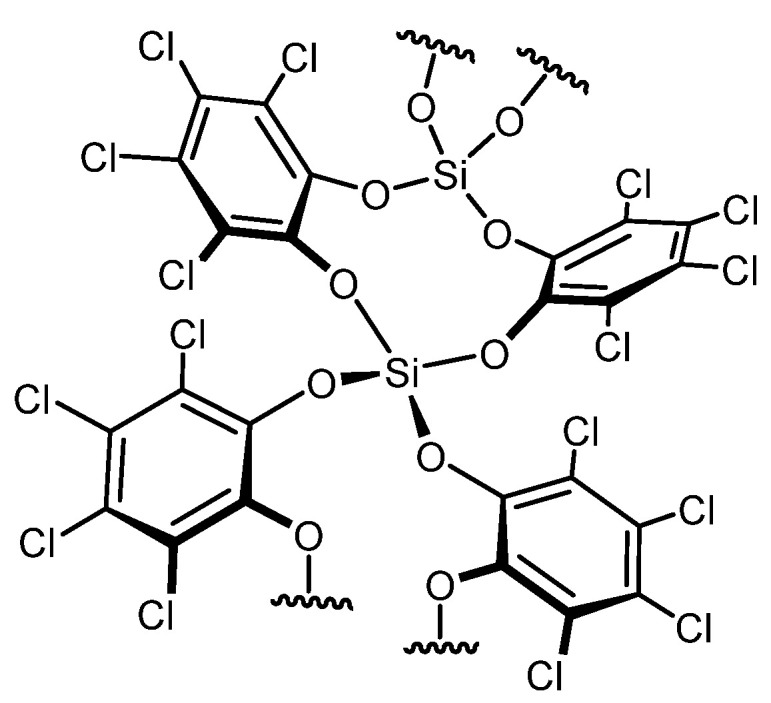
Lewis superacid bis(perchlorocatecholato)silane as a catalyst for C-O bond metathesis of polyethers [150].

Polyethers are widely valued for their exceptional chemical and physical properties, such as low-temperature flexibility and resistance to heat and oils [147]. These characteristics make them essential reagents in polymer manufacturing, fueling ongoing efforts to develop innovative and environmentally friendly synthetic methodologies [148]. Alongside synthesis, significant advancements have been achieved in the depolymerization and recycling of polyethers, including strategies based on ring-closing metathesis reactions [149]. For example, N. Ansmann and colleagues [150] investigated the mechanism of silicon-catalyzed C−O bond ring-closing metathesis in polyethers, identifying a second-generation silicon Lewis superacid as an effective catalyst for this transformation, as illustrated in Scheme 10.

The mechanism was clarified through DFT computational studies, revealing that the catalyst’s low tendency for chelation and deactivation by polyethers, such as polyethylene glycol, is a key advantage. In contrast, other Lewis acids like Fe(OTf)_3_ and AlCl_3_ exhibited mono-, bi-, and tridentate binding with diglyme, adversely affecting product conversion.

**Figure 11:**
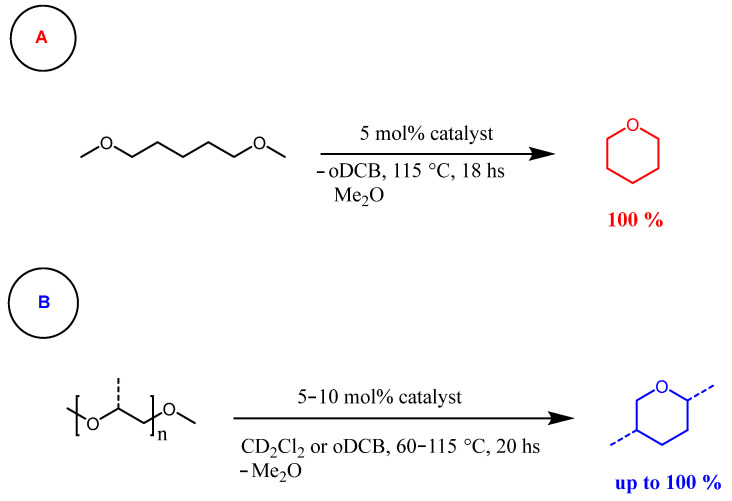
Adapted from Ansmann et al., Angew. Chem. Int. Ed. 2023, 62, e202210132. © 2023 Wiley-VCH GmbH. Adapted with permission. Reaction scheme of the Lewis acid catalyzed selective degradation of 1,5-dimethoxypentane (**A**) and selective ring closing C-O bond metathesis of polyethylene glycol and polypropylene glycol (**B**) [150].

Figure 11 illustrates the reaction scheme starting from 1,5-dimethoxypentane (Figure 11A) or polyethylene glycol/polypropylene glycol (Figure 11B), along with the corresponding optimized reaction conditions.

**Scheme 11:**
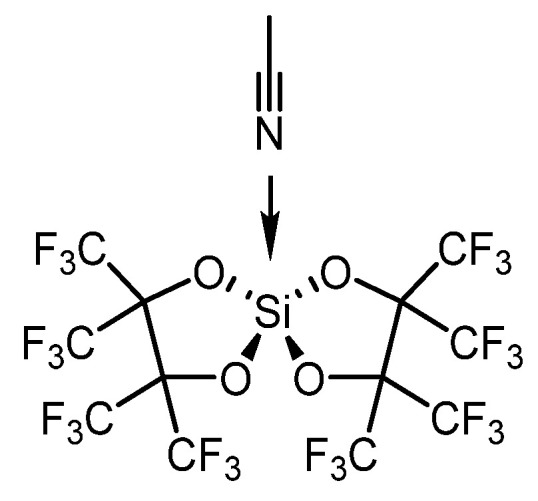
Acetonitrile-adduct of a Lewis superacidic silane (Si(pinF)2·MeCN) as a catalyst for C-O bond metathesis of polyethers [151].

In a recent work, Florian S. Tschernuth et al. [151] investigated the interesting catalytic activity of a Lewis superacidic bis(perfluoropinacolato) silane for the degradation of aliphatic ethers. The catalyst is reported in the following Scheme 11.

Ring-closing metathesis is typically affected by the availability of two or three coordination sites on the catalyst, leading to coordination and subsequent deactivation [152]. However, catalysts with blocked or less favorable multiple-coordination sites, such as the acetonitrile-adduct of a Lewis superacidic silane (Si(pinF)_2_·MeCN), demonstrated enhanced activity. This study revealed that the coordinated acetonitrile is exchanged with the etheric substrate used. Table 11 summarizes the optimized reaction conditions and corresponding products for the degradation of 1,5-dimethoxypentane (1,5-DMP), diglyme, poly(ethylene glycol) dimethyl ether (PEG DME), and 18-crown-6 [151].

## 5. Summary and Outlook

This review examines innovative catalytic technologies for the depolymerization of key polymers of significant industrial and environmental interest. We focus on cellulose, lignin, polyester plastics, polyamides, polyurethanes, and polyethers. Cellulose is highlighted as a primary source of glucose and other organic platform molecules, which serve as building blocks for high-value materials across pharmaceutical, food, energy, and various other sectors. Lignin, traditionally derived from petroleum-based resources, is recognized as an important renewable source of aromatic and aliphatic compounds and has been extensively studied recently, particularly for applications in biofuels and building materials. The review also addresses the depolymerization of the most widely used plastics worldwide, polyesters, polyamides, polyurethanes, and polyethers, primarily produced from fossil feedstocks. We demonstrate how sustainable catalytic methodologies can efficiently recycle these polymers into valuable monomers and intermediates, enabling their reuse in diverse applications. Amid the ongoing ecological transition, heightened awareness of environmental challenges has underscored the importance of recycling strategies over conventional disposal methods. In this context, catalysis plays a pivotal role in enhancing process efficiency and scalability at the industrial level. The development of mild, non-destructive techniques to obtain monomers, facilitating multiple recycling cycles without compromising product quality, represents a promising frontier in sustainable polymer science.

### Future Challenges

Development of cost-effective and environmentally sustainable catalytic systems. For an innovative process to be successfully scaled up, it must achieve an optimal cost–benefit balance. Many catalysts are expensive materials, and only non-destructive strategies under mild conditions can enable true recycling cycles. Mild reaction conditions are also closely linked to energy efficiency and cost savings. Additionally, solvent-based processes should be minimized or eliminated where possible, as this can significantly reduce costs and environmental impact. The challenge lies in identifying catalytic systems that not only reduce the environmental footprint but also remain economically viable across different industrial applications and polymer types.

For the production of high-purity monomers with minimal by-products and high atom economy, a method is truly sustainable only if it ensures high yield and selectivity. Achieving this requires growing interest and investment in this field of research to drive further advancements. The development of highly selective catalysts capable of breaking down complex polymer matrices into uniform, reusable monomers remains a key hurdle. Strategies that enhance atom economy and suppress unwanted side reactions are essential to make chemical recycling truly competitive with mechanical recycling and incineration.

For the design of simple, easily scalable processing plants, beginning with straightforward laboratory reactors and processes increases the likelihood of successful scale-up beyond the lab. However, the real challenge is translating these lab-scale methodologies into pilot and industrial-scale systems without compromising efficiency, safety, or sustainability. Modular reactor design, process intensification, and integration with existing industrial infrastructure will be critical to facilitate large-scale deployment.

For the integration with renewable energy sources and digital technologies, ss sustainability becomes increasingly central, coupling depolymerization processes with renewable energy, such as solar or wind, can help further reduce carbon emissions. Additionally, the integration of digital tools such as AI and machine learning can assist in catalyst design, process optimization, and real-time monitoring, significantly enhancing overall system performance and reliability.

Addressing the heterogeneity of post-consumer plastic waste, one of the most complex challenges is the treatment of mixed and contaminated waste streams. Real-world plastic waste is rarely uniform, often containing additives, dyes, and mixed polymers that complicate depolymerization. Developing adaptable and robust catalytic systems capable of efficiently processing such heterogeneous materials without extensive pre-treatment remains a major obstacle.

For regulatory, economic, and societal adoption, beyond technical challenges, broader systemic issues also need to be addressed. Regulations must evolve to support innovative recycling technologies, including incentives for industries to adopt chemical recycling. Moreover, consumer awareness and willingness to participate in circular economy initiatives are critical. Public policy, education, and transparent lifecycle assessments will be essential to build trust and drive widespread adoption.

The circular economy, if implemented sustainably, holds significant potential as a future strategy to minimize the waste of both natural and synthetic resources. Meeting these challenges will require a multidisciplinary approach involving collaboration between chemists, engineers, industry stakeholders, policymakers, and society at large.

## Figures and Tables

**Table 1:** Sources of cellulose with their Mean DPw peak values for cello-oligomers and Percentage change of DPw.

Source of Cellulose	Mean DPw Peak 0 min and 120 min		Percentage Change of DPw ΔDPw (%)
Sawdust	351	72	79.5
Sago pith wastes	564	30	94.7
Corn Cob	329	88	78.1
Sugarcane bagasse	571	37	93.5

**Table 2:** Cellulose conversion in molten salt hydrates solutions with catalysts.

Substrate	MSH/Catalyst	Main Products (%Yield)	Ref.
MCC	Beta and ZSM-5 zeolites with SiO_2_/Al_2_O_3_ = 30 and 50	Gluco oligomers (54.4%), glucose (28.3%) & HMF (0.2%)	[52]
MCC	ZnCl_2_ (72 wt%)/HCl (0.2 M)	HMF (69.5%)	[57]
Cellulose	ZnCl_2_·3H_2_O/SO_4_/TiO_2_	Gluco oligomers (9.4%), glucose (50.5%), HMF (3.4%), fructose (5.9%), & LA (5.1%)	[58]
MCC	LiBr (55 wt%) Activated/Activated carbon	Glucose (80%) & LA (4%)	[59]

**Table 3:** Mechanocatalytic depolymerization of cellulose in the presence of different solid acid catalysts.

Catalyst	H^+^ Exchange Capacity (mmol/g)	Solubility (%)
Blank	-	<5
Aquivion PW98	1.0	90
SBA–SO_3_H	0.2	60
CMK-3-SO_3_H	0.7	87
Kaolinite	-	50
Aquivion PW66	1.45	99
Aquivion PW79	1.26	32
Aquivion PW87	1.15	80

**Table 4:** Zirconia and other grinding balls with their properties.

Material	Diameter (mm)	Density (g/cm^−3^)
Zirconia	3	5.68
Stainless steel	2	7.8
Stainless steel	4	7.8
Tungsten carbide	3	15.63

**Table 5:** Catalysts and products yield by mechanocatalytic process ^a^.

Catalyst	Products Yield (%)
HNbMoO_6_	14
kaolinite	4
NiO	0.3
SnO_2_	0.6
TiO_2_	0.5
Nb_2_O_5_	0.9
H-Montmorillonite	3
USY zeolite	3
Mg–Al HT	0

^a^ Yields refer to reaction stopped after 4 h.

**Table 6:** Summary of homogeneous catalytic approaches for lignin depolymerization.

Catalysis Type	Advantages	Limitations	References
Homogeneous Acid Catalysis	High reactivity, low cost	Low selectivity, harsh conditions, byproduct formation, corrosivity, toxic reagents	[78,79,80,81]
Homogeneous Base Catalysis	High efficiency at moderate temperatures, potential for vanillin production	Solvent dependency, limited performance in aqueous media	[87]
Combined Acid–Base Catalysis	Effective stabilization of intermediates, dual activation	Requires careful optimization of multi-functional catalysts	[88]
Homogeneous Metal Catalysis	High activity and selectivity, tunable ligands, reusable on supports	High cost, elevated T/P needed, sensitivity to reaction conditions	[95,96]

**Table 7:** Summary of heterogeneous catalysis for lignin depolymerization.

Catalysis Type	Advantages	Disadvantages	References
Heterogeneous–Solid Acid	Regenerable, high conversion, environmentally sustainable	Complex selectivity; possible char formation	[97,98,99,100,101,102,103,104,105]
Heterogeneous–Metal-Supported	High activity, large surface area, good selectivity, reusable	Sensitive to metal type and support; potential for deactivation	[106,107,108,109,110,111,112,113,114,115]

**Table 8:** Reaction optimization for the acidolysis depolymerization of PET [a].

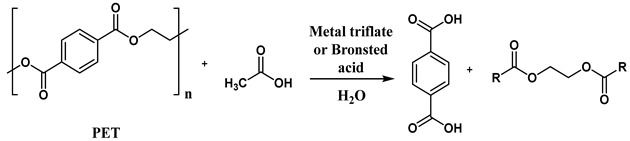				
Catalyst	T (°C)	C_cat_ (M)	C_H20_ (M)	Yield (%)
Hf(OTf)_4_	180	0.25	0	60
Hf(OTf)_4_	180	0.25	0.5	72
Fe(OTf)_3_	180	0.25	0.5	64
Al(OTf)_3_	180	0.25	0.5	60
TfOH	180	0.25	0.5	98
Tf_2_NH	180	0.25	0.5	58

[a] Reactions conditions: 0.5 mmol PET was put in 0.2 mL of Cat./H_2_O/AcOH solution in a capped vial at 180 °C for 40 min.

**Table 9:** Reaction optimization for the dimethyl carbonate-aided methanolysis depolymerization of PET [a].

Catalyst	DMC (mL)	MeOH (mL)	DMT (%)	EC (%)
LiOMe	1.5	0.2	83	70
KOMe	1.5	0.2	95	93
NaOMe	1.5	0.2	95	86
NaOMe [b]	1.5	0.2	93	92
NaOMe	1	0.13	98	67
NaOMe	0.5	0.065	93	60

[a] PET textile (100 mg), catalyst (5 mol % based on the PET alternating unit), DMC (1.5 mL), MeOH (0.2 mL), 50 °C, and 2 h. Abbreviations. DMC: dimethyl carbonate, DMT: dimethyl terephthalate, EC: ethylene carbonate. [b] Reaction time: 1 h.

**Table 10:** Reaction optimization for the depolymerization of low molecular weight polyamide 66 using Ru pincer complex [a].

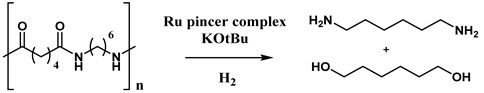			
T (°C)	P (H_2_) Bar	Yield (%) Diamine	Yield (%) Diol
150	70	12	<5
180	100	60	35
200	100	78	62
200	80	70	47

[a] Low-molecular-weight polyamide (0.3 g, 1.25 mmol according to the repeating unit of polyamide 66), Ru pincer complex as catalyst (0.01 mmol), KO*t*Bu (0.04 mmol), THF (5 mL).

**Table 11:** Reaction optimization for the degradation of 1,5-dimethoxypentane (1,5-DMP), diglyme, poly(ethyleneglycol)dimethylether (PEG-DME), and 18-crown-6 [a].

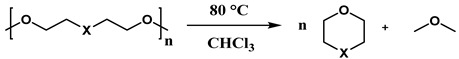			
Substrate	Catalyst (%mol)	Time (h)	Yield (%)
1,5 DMP	1–5	1–20	96–97
Diglyme	1–5	2.5–96	96–99
18-crown-6	30	30	88
PEG-DME	225	18	87

[a] %Yield of tetrahydropyran and 1,4-dioxane was conducted by ^1^H NMR using mesitylene as an internal standard.

## Data Availability

Not applicable.
